# Fitting the Elementary Rate Constants of the P-gp Transporter Network in the hMDR1-MDCK Confluent Cell Monolayer Using a Particle Swarm Algorithm

**DOI:** 10.1371/journal.pone.0025086

**Published:** 2011-10-18

**Authors:** Deep Agnani, Poulomi Acharya, Esteban Martinez, Thuy Thanh Tran, Feby Abraham, Frank Tobin, Harma Ellens, Joe Bentz

**Affiliations:** 1 Department of Biology, Drexel University, Philadelphia, Pennsylvania, United States of America; 2 Preclinical Drug Metabolism and Pharmacokinetics, GlaxoSmithKline, King of Prussia, Pennsylvania, United States of America; 3 Scientific Computing and Mathematical Modeling, GlaxoSmithKline, King of Prussia, Pennsylvania, United States of America; The Scripps Research Institute, United States of America

## Abstract

P-glycoprotein, a human multidrug resistance transporter, has been extensively studied due to its importance to human health and disease. In order to understand transport kinetics via P-gp, confluent cell monolayers overexpressing P-gp are widely used. The purpose of this study is to obtain the mass action elementary rate constants for P-gp's transport and to functionally characterize members of P-gp's network, i.e., other transporters that transport P-gp substrates in hMDR1-MDCKII confluent cell monolayers and are essential to the net substrate flux. Transport of a range of concentrations of amprenavir, loperamide, quinidine and digoxin across the confluent monolayer of cells was measured in both directions, apical to basolateral and basolateral to apical. We developed a global optimization algorithm using the Particle Swarm method that can simultaneously fit all datasets to yield accurate and exhaustive fits of these elementary rate constants. The statistical sensitivity of the fitted values was determined by using 24 identical replicate fits, yielding simple averages and standard deviations for all of the kinetic parameters, including the efflux active P-gp surface density. Digoxin required additional basolateral and apical transporters, while loperamide required just a basolateral tranporter. The data were better fit by assuming bidirectional transporters, rather than active importers, suggesting that they are not MRP or active OATP transporters. The P-gp efflux rate constants for quinidine and digoxin were about 3-fold smaller than reported ATP hydrolysis rate constants from P-gp proteoliposomes. This suggests a roughly 3∶1 stoichiometry between ATP hydrolysis and P-gp transport for these two drugs. The fitted values of the elementary rate constants for these P-gp substrates support the hypotheses that the selective pressures on P-gp are to maintain a broad substrate range and to keep xenobiotics out of the cytosol, but not out of the apical membrane.

## Introduction

P-glycoprotein (P-gp) is a member of the ATP binding cassette (ABC) family of proteins that has been extensively studied because of its ability to render cells resistant to many chemotherapeutic agents and for causing clinically important drug-drug interactions [1], [2], [3], [4]. A molecular understanding of P-gp activity requires both structural knowledge [5], [6], [7], [8] and functional knowledge of transport kinetics in physiologically relevant systems [9], [10], [11], [12], [13], [14]. Confluent cell monolayers are widely used as models for human tissues in which P-gp is expressed [14], [15], [16], [17], [18], [19]. Here, we use a confluent monolayer of hMDR1-MDCKII cells to develop a functional description of the P-gp associated multi-transporter network by obtaining elementary rate constants that regulate the flow of several P-gp substrates between apical and basolateral compartments. 

**Figure 1:**
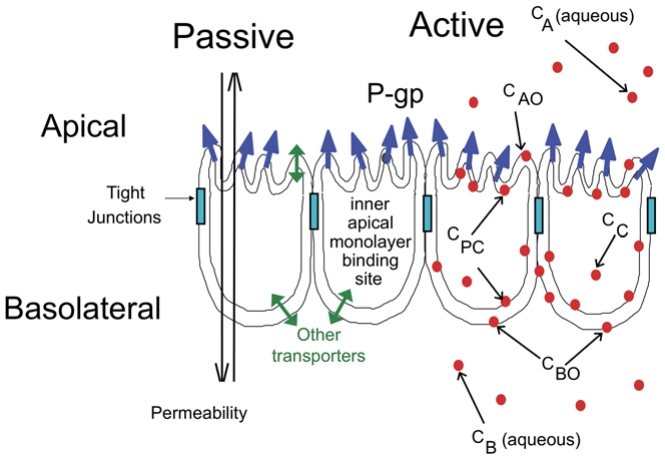
Model of the Confluent Monolayer of Polarized Cells. Model of a confluent cell monolayer, with the apical membrane on top and the basolateral membrane below, where it binds to the polycarbonate insert. P-gp expressed on the apical membrane transports substrate from the inner apical membrane monolayer into the apical chamber. The concentration of substrate in the apical and basolateral chambers, C_A_ and C_B_, are measured, while the concentration of substrate in the inner plasma membrane, C_PC_, and the cytosol, C_C_, are predicted as part of the mass action modeling and data fitting process. Some compounds use other transporters expressed by the MDCKII-hMDR1 confluent cell monolayer. Passive permeability occurs in both directions.

Figure 1 shows the basic transport pathways across a confluent cell monolayer. There is partitioning of substrates into the membranes they face; passive permeability across the lipid bilayers; facilitated transport across both the basolateral and apical membranes; and both primary and secondary active transport. P-gp's primary active transport across the apical membrane is modeled using Eq (1), the standard Michaelis-Menten reaction
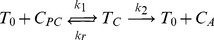
(1)where *T_0_* is the empty transporter, *C_PC_* is the concentration of substrate in the inner apical membrane, *T_1_* is the transporter bound to substrate, and *C_A_* is the substrate after efflux into the apical compartment. P-gp's ATPase activity is not measured within a confluent cell monolayer, but is required for efflux from P-gp into the apical compartment [20], [21], [22]. However, we have shown that P-gp efflux rates are the same at the beginning of an experiment and 3 hr later, so the required ATP levels are being maintained throughout the 4–6 hr experiment [23].

Typically, the kinetic analysis of transcellular transport uses some form of Michaelis-Menten steady state equations [2], [10], [12], [14], [16], [24], [25], [26]. While these equations can often fit the efflux data for confluent cell monolayers, the fitted *V_max_* and *K_M_* parameters are complex convolutions of the elementary rate constants. We showed this using simulated transport kinetics from our mass action model for the confluent cell monolayer. We analyzed the model data using Michaelis-Menten steady state equations [27]. The basic conclusion was that the value of the fitted Michaelis constant *K_M_* to the simulated data was not correlated with the standard value of *K_M_* = (*k_2_*+*k_r_*)/*k_1_*, from the elementary rate constants used to create the simulations in the first place. Thus, two experimentally fitted *K_M_* values that are close to one another numerically could come from original (*k_2_*+*k_r_*)/*k_1_* values that differ by as much as 3 orders of magnitude. This is the consequence of the convolution of all the kinetic parameters that drive P-gp transport into just a single “*K_M_*”, which has a small probability of predicting the *in vivo* situation. We believe that the elementary rate constants will extrapolate in vivo much more successfully.

The need to know the elementary rate constants extends to the basic IC_50_ analysis of transport. For the confluent cell monolayer, when the dissociation constant of the inhibitor to P-gp is denoted *K_I_*, we have shown that the ratio of the IC_50_/*K_I_* increases with increased P-gp surface density and probe-substrate elementary efflux rate constants and decreases with the contributions of other probe-substrate transporters [28]. The simple IC_50_ analysis is very different with confluent cell monolayers, or tissue, than it is with water soluble enzymes, upon which the standard IC_50_ equation were tested [28].

Obviously, obtaining these elementary rate constants is a difficult fitting problem requiring robust numerical approaches. Our previous fittings were accomplished using a hierarchical approach, with several fitting steps being manual [23], [29], [30]. It's limitations were that it could analyze only one drug concentration at a time per computer processor, the collation of the consensus rate constants had to be done manually, yielding broad ranges of “equivalent best fits” which changed as new datasets were examined and, worse, about a third of the datasets failed to yield convergent answers with the algorithm for no apparent reasons. These were serious limitations.

To overcome these problems, we have developed two major computational refinements: 1) a new fitting program that determines the elementary rate constants simultaneously from all relevant data sets, which can run serially or in parallel; and 2) the use of a global optimization package based upon the Particle Swarm algorithm [31], which proved to be far faster and more accurate. The combination of these two refinements provides robustness, i.e. all the applicable data can be fitted simultaneously. A comparison of the two fitting algorithms showed that the average coefficient of variation per fitted data set, <CV/dataset> is about 35% smaller using the Particle Swarm algorithm and fits were completed about 20-fold faster.

The fitting of all data has led to significant changes from previous estimates for kinetic parameters and P-gp efflux active surface density. The fitted values of the kinetic parameters still make sense with respect to the hypothesis that the primary selective pressure on P-gp to respond to all of xenobiotics, many of which it is encountering for the first time. The older algorithm supported the same hypothesis [23].

## Materials and Methods

### Experimental

P-gp substrates, inhibitors, cell line and culture conditions have previously been described [23], [29], [30]. Briefly, Madin-Darby Canine Kidney cell line overexpressing human MDR1 (MDCKII-hMDR1) was purchased from the Netherlands Cancer Institute (NKI, Amsterdam, Netherlands). Cells were split twice a week and maintained in culture media (DMEM supplemented with 10% Fetal Bovine Serum, 50 units/ml penicillin and 50 µg/ml streptomycin). Cells were kept at 37°C in 5% CO_2_.

P-gp mediated transport was measured in 12-well transwell Costar plates fitted with polycarbonate membrane inserts. Cells were seeded at a density of 175,000–200,000 cells per insert and grown for four days in culture media. Cells were given fresh media one day after seeding. Prior to the experiment, culture media was removed and cells were preincubated for 30 minutes with either transport medium alone (see above) or transport medium supplemented with 2 µM GF120918 to inhibit P-gp. Transport of a range of concentrations of amprenavir, loperamide, quinidine and digoxin across the confluent monolayer of cells was measured in both directions, i.e. apical to basolateral (A>B) and basolateral to apical (B>A) in the presence and absence of GF120918. For incubations in the presence of GF120918, the inhibitor was added to both chambers. 0.5 µCi/ml of ^3^H-amprenavir, ^3^H-quinidine, ^3^H-loperamide, or ^3^H-digoxin was added to each respective drug concentration to allow quantitation of transport from donor to receiver chambers by liquid scintillation counting. In addition, 0.25 µCi/ml ^14^C-mannitol or 100 µM Lucifer yellow was added to monitor cell monolayer integrity. At the indicated time points, 25 µL samples were taken from both donor and receiver chambers, mixed with 10 ml of Ultima Gold scintillation cocktail and counted using a Hewlett Packard Liquid Scintillation Counter or using the Perkin Elmer TopCount [23], [29], [30]. While the data for amprenavir, quinidine and loperamide were obtained in a single 4–6 hour experiment, a step-wise development of the digoxin data was used for the Particle Swarm fitting algorithm to determine the kinetic parameters. This has been explained in the supporting material (Text S1 and Figures S1, S2, S3 and S4).

### Fitting of data sets using particle swarm

We adapted the particle swarm program [31] to fit the elementary rate parameters for our data sets. The program is written in MATLAB (Natick, MA), using ODE23s numerical integrator, since other numerical integrators deviate at the long time points. The “goodness of fit” to a particular drug data set was quantified by the coefficient of variation, <CV/dataset>, defined as the standard deviation between the data and the simulated fit divided by the initial drug concentration. This normalizes the comparisons of fits over different initial drug concentrations. The fit for a particular drug would be quantified by the average of all CV/dataset for that drug. The fittings and most simulations were performed on a 24-Microway processor cluster at the Department of Scientific Computing and Mathematical Modeling, GSK, Upper Merion, PA. Drug equilibrium partition coefficients were obtained previously [23].

Briefly, the program starts with a user assigned number of particles that are randomly deployed over the entire multi-dimensional parameter space, within user assigned upper and lower bounds, unless otherwise noted. The particles are allowed to randomly explore the entire parameter space. Each particle reports its coordinates and CV to the manager processor, which determines the particle with the lowest CV and then randomly reassigns the particles to new positions, with a small bias toward to coordinates of the current minimum CV. This dual particle and swarm memory is used to not only stochastically explore the parameter space, but to converge to the global minimum. This particular version of the many implementations of particle swarm has the advantage that it includes an additional step of local polling of the objective function, which allows the particle to be moved out of a local minimum. The particle swarm approach searches all the dimensions simultaneously, so there are no implicit biases in the search for the global minimum. The process stops when either all of the particles have converged to the same global minimum or when the number of function evaluations exceeds a pre-assigned maximum.

## Results

**Figure 2:**
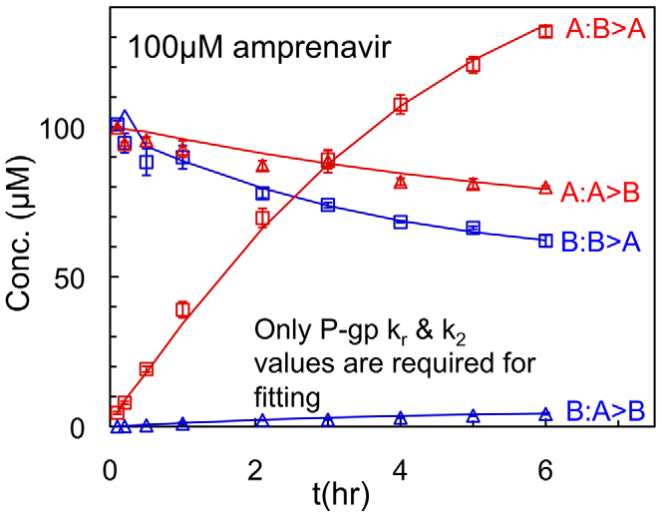
Amprenavir transport over 6 hours across the MDCKII-hMDR1 cell monolayer. Amprenavir transport A>B and B>A over 6 hours across the MDCKII-hMDR1 confluent cell monolayer with 100 mM on the donor side initially. The symbols show the data points with error bars showing the standard deviation of triplicate measurements. A∶B>A denotes the substrate concentration in the apical chamber when the basolateral chamber is the donor, while B∶B>A denotes the substrate concentration remaining in the donor basolateral chamber. The A∶B>A transport is high because P-gp actively pumps drug into the receiver apical chamber. The B∶A>B denotes the substrate concentration in the basolateral chamber when the apical chamber is the donor, while A∶A>B denotes the substrate concentration remaining in the donor apical chamber. The B∶A>B transport is low because P-gp actively pumps drug back into the donor apical chamber. The lines show the best fits for amprenavir transport assuming there are no other transporters except P-gp.

We start by showing the outcome of one fitting, out of a total of 72, in order to explain the amount and quality of the data being fitted and how the fitting algorithm evolved. Fig. 2 shows the fit for 100 mM amprenavir. The amprenavir concentrations used were larger than those used for the other drugs because it has the weakest binding constant to P-gp and the fastest efflux rate constant from P-gp that we have measured to date [32]. So relatively large concentrations are needed to reach saturating levels, i.e. curvature in the transport curve within 3 hrs of transport. This curvature shows that the system is reaching steady-state, where the P-gp efllux out of the cells into the apical chamber equals the passive permeability and facilitated transport into the cell from the apical chamber.

Each data set has 4 curves to be simultaneously fitted. There is the time course for the donor and receiver chambers for B>A transport: B∶B>A (the probe-substrate concentration in the basolateral chamber when the basolateral chamber is the donor) and A∶B>A (the probe-substrate concentration in the apical chamber when the basolateral chamber is the donor), i.e. the amprenavir concentration in the donor chamber and in the receiver chamber, respectively. There is also the time course for the donor and receiver chambers for A>B transport: A∶A>B (the probe-substrate concentration in the apical chamber when the apical chamber is the donor) and B∶A>B (the probe-substrate concentration remaining in the basolateral chamber when the apical chamber is the donor), respectively. The 6 min time point establishes a measured initial concentration in both compartments.

All data sets have 9 time points ending at 4 or 6 hours, depending upon how long it took to achieve adequate curvature in the data. The error bars are the standard deviation from triplicate wells. Thus, the average of triplicates yields 36 data points within each dataset. The solid line shows the fit using the fitted kinetic parameters shown below in Table 1. All of the amprenavir data have been fitted with just two drug specific numbers: the dissociation rate constant from P-gp back to the membrane, *k_r_*, and the efflux rate constant from P-gp into the apical chamber, *k_2_*. The drug independent numbers for the fitted values of the P-gp efflux active surface density, *T(0)*, the association rate constant from the membrane to P-gp, *k_1_*, were simultaneously fitted using all drug datasets, as shown below.

**Table 1:** Averages and standard deviations of transport parameters for the 24 independent replicate fits.

Substrate	Associationto P-gp*k_1_* (M^−1^ s^−1^)^a^average±sd{range}	Efflux ActiveP-gp Surface Density[P-gp](per mm^2^)^b^	Dissociation from P-gpTo Apical Bilayer*k_r_* (s^−1^)^c^	Efflux toApical Chamber*k_2_* (s^−1^)^d^	Partition Coeff.^e^K_PC_	Binding Constant toP-gp from Inner Apical Membrane*K_C_* (M^−1^)^f^ = *k_1_*/*k_r_*	Passive Permeability Coefficient atsteady-state^g^(nm/sec)		Other Bidirectional Transporter^h^(s^−1^)	
							*P_BA_*	*P_AB_*	*k_B_*	*k_A_*
Amprenavir(n = 19)	(1±0.4)×10^+8^{(0.6–2)×10^+8^}	800±200{500–1300}	(2±0.8)×10^+5^{(0.9–4)×10^+5^}	30±8{17–45}	200±20	600±100{400–900}	420±50	350±30	0	0
Digoxin(n = 4)	Same asabove	Same as above	(3±1)×10^+4^{(2–7)×10^+4^}	3±1{1–6}	100^i^	3,000±200^j^{2700–3300}	50±10	40±10	40±3{35–45}	40±20{20–95}
Loperamide(n = 31)	Same asabove	Same as above	(4±2)×10^+4^{(2–7)×10^+4^}	0.4±0.08{0.2–0.5}	3,000±600	3,000±400{2000–4000}	320±90	320±70	100±7{90–120}	0
Quinidine(n = 16)	Same asabove	Same as above	(8±4)×10^+^{(4–20)×10^+3^}	3±0.4{1–4}	700±30	(1±0.2)×10^+4^{(0.9–2)×10^+4^}	670±50	670±50	0	0

a *k_1_* is the drug independent association rate constant from the membrane to P-gp. The average value±standard deviation for the 24 independent replicate fits obtained for all drugs is shown, while the entire range is shown in curly brackets, Fig. 3C.

b *T(0)* is the surface density of efflux active P-gp in the apical membrane inner monolayer for all drugs. The average value±standard deviation for the 24 independent replicate fits obtained for all drugs is shown, while the entire range is shown in curly brackets, Fig. 3C. The units P-gp/mm^2^ can be converted to mmols P-gp per liter of inner apical membrane simply by dividing by 0.8 [23].

c *k_r_* is the dissociation rate constant from the P-gp binding site into the apical bilayer. The average value±standard deviation for the 24 independent replicate fits obtained for all drugs is shown, while the entire range is shown in curly brackets, Fig. 4A.

d *k_2_* is the efflux rate constant from the P-gp binding site into the apical chamber. The average value±standard deviation for the 24 independent replicate fits obtained for all drugs is shown, while the entire range is shown in curly brackets, Fig. 4A.

e The partition coefficient between the cytosol and the inner plasma/apical monolayer, *K_PC_* [23]. Cell membrane partition coefficients were estimated using 0.1 mm extruded unilamellar liposomes (LUV) whose lipid compositions mimic roughly the lipid compositions of the respective membrane monolayers: inner cytosolic PS/PE/chol (1∶1∶1); apical outer, PC/SPH/chol; and basolateral outer, PC/chol (2∶1). Only the inner cytosolic partition coefficient, *K_PC_*, is shown in this table.

f *K_C_* = *k_1_*/*k_r_* is the substrate binding constant from inner apical membrane monolayer to P-gp. The average value±standard deviation for the 24 independent replicate fits obtained for all drugs is shown, while the entire range is shown in curly brackets, data not shown. This value is calculated from the actual fitted values, rather than the average 1-digit values of *k_1_* and *k_r_* reported in the Table.

g *P_BA_* and *P_AB_* refers to the +GF120918 steady-state passive permeability coefficient, B>A and A>B respectively. These values increase initially to a final steady-state value [32], which is reported here as an average value±standard deviation over all relevant datasets.

h *k_B_* and *k_A_* refers to the 1^st^ order rate constant for transport through a bidirectional transporter for digoxin and for loperamide. The average value±standard deviation for the 24 independent replicate fits obtained for all drugs is shown, while the entire range is shown in curly brackets, Fig. 4B.

i Digoxin's partition coefficients have not yet been measured. We set it to 100, as that is the lower bound for measured values.

### Fitting the kinetic parameters by Particle Swarm

We had a large number of drug data sets that had to be fitted simultaneously. For each drug, the number of data sets and drug concentrations varied. For the data in Tran et al. [23] there was: amprenavir with 5 datasets and concentrations varying from 50–150 µM; quinidine with 6 datasets and concentrations varying from 1–10 µM; and loperamide with 8 datasets and concentrations varying from 0.1–1 µM. This yields 19 datasets. For the data in Acharya et al. [29], [30] there were: amprenavir with 14 datasets and concentrations varying from 20–100 µM; quinidine with 10 datasets and concentrations varying from 0.1–10 µM; loperamide with 25 datasets and concentrations varying from 0.01–30 µM; and digoxin with 4 datasets and concentrations varying from 10–50 µM. This yields 53 datasets. All together there were 72 datasets, each with 36 data points over time to be fitted, i.e. 2592 total data points. The point is that there are far more data points to be fitted simultaneously than the 13 parameters we eventually fit here.

For all drugs, the highest concentrations yielded nearly saturated P-gp binding, so that P-gp mediated transport was a small contribution to the net passive flux. The smallest concentrations yielded fairly linear curves due to sparse P-gp binding. Overall, the entire dynamic range of transport for each drug was covered, allowing each of the rate constants to be measured. In other words, there was no single step that was rate-limiting at all drug concentrations. This is why all rate constants could be fitted and why the Michaelis-Menten steady-state equations do not yield *K_M_* values correlated with the elementary rate constants [27].

### Fitting the drug independent values: *T(0)* and *k_1_*

Previously we found that the fitted total P-gp surface density, *T(0)*, was drug independent [23]. Since each dataset was fitted separately in the old algorithm, a *T(0)* was fitted for each dataset and we found that they clustered together. That was a benchmark for our fitting approach, since there is only one species of P-gp. By the old approach we also found that the association rate constant, *k_1_*, was drug independent [23], which made sense if the entry to the P-gp binding site is large compared to the molecular sizes of the drugs we studied [8].

Our first step here was to determine whether the Particle Swarm algorithm would show that *T(0)* and *k_1_* could be fitted to consensus values for all of the drug data we had. We assumed that all drugs had a *k_r_* and *k_2_* for P-gp, Eq. 1. Amprenavir and quinidine required no other transporters. We verified that loperamide required a basolateral transporter and digoxin required both a basolateral transporter and an apical transporter, see supporting material (Text S1 and Table S1) [29].

Preliminary separate fits of the data of Tran et al. [23] and Acharya et al. [29], [30] showed no significant difference in the fitted parameters. So, all the datasets were simultaneously fitted for *T(0)* and *k_1_*. The drug specific kinetic parameters were fitted using just the specific drug datasets, e.g. the digoxin specific kinetic parameters were fitted using only the digoxin datasets.

To estimate the uncertainty of the fits for *T(0)* and *k_1_*, we used a Monte Carlo approach by running 24 independent replicate fittings. This would yield 24 independent {*T(0)*, *k_1_*} pairs of optimal fits, each of which had an associated vector of the other drug-specific rate constants. If the fitting surface were a smooth “funnel”, we would expect all replicate fits would come to roughly the same point. This was not the outcome, but the ranges we found were tight enough to yield robust estimates for both the fitted parameters and the standard deviation of the fitted parameters.

Preliminary fittings showed that the upper bound for the concentration of efflux active P-gp, *T(0)*, had to be set to 2.5×10^−3^(M) within the inner apical membrane, which would be equivalent to P-gp occupying about 25% of the efflux active apical plasma membrane surface. This would be too high for a final answer, but it is acceptable as an upper bound. Reducing the upper bound led to some clustering of intermediate fits near this upper bound, which must be avoided. The upper bound for the association rate constant, *k_1_*, was set at 1×10^9^ (M^−1^ s^−1^), which would be in the range of lipid lateral diffusion control [23]. The lower and upper bounds used for all of the fits are shown by the range of the x- and y-axes in the figures. Lower bounds were always well below the fitted values.

We found that the fittings needed to be done in sequential rounds. The first round result for {*T(0)*, *k_1_*} is shown in Fig. 3A, where the axes show the upper and lower bounds for the fitting. The empty triangles show the outcome for each of the 24 replicate fittings. All individual fits cluster near, but not at, the upper bounds for both parameters. The average values over the 24 independent fits of the log_10_{*T(0)* (M)} = −2.9±0.3 and log_10_{*k_1_* (M^−1^ s^−1^)} = 8.1±0.4, shown by the solid triangle, with standard deviations shown by the error bars. The average <CV/dataset> = 0.03, over all 72 datasets, while the old algorithm gave about <CV/dataset > = 0.04. This is a 30% improvement in the average fit quality and the fitted rate constants are quite different from the old algorithm.

**Figure 3:**
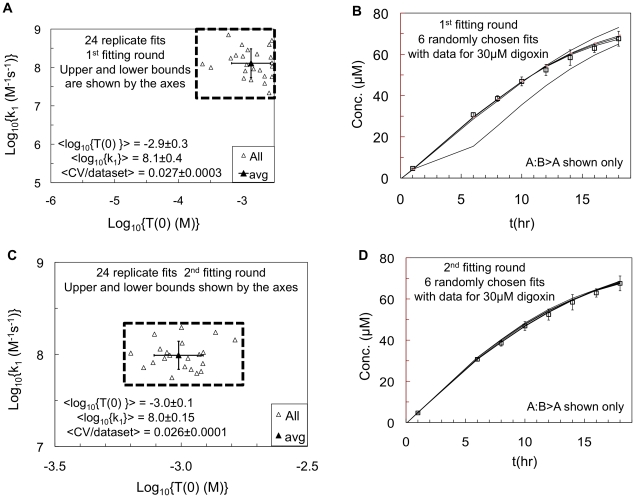
Simultaneous fits of P-gp efflux active surface density, T(0), and association rate constant, k_1_. 24 independent replicate fits of all 72 experimental data from Tran et al. [23] and Acharya et al. [29], [30]. All 13 kinetic parameters were simultaneously fitted to all relevant datasets. For all figures, the x- and y-axes show the user-fixed lower and upper bounds used in each fitting round. **Fig. 3A** shows the 1^st^ round of fitting for the drug independent values of the surface density of efflux active P-gp in the apical membrane, *T(0)*, and the association rate constant *k_1_*. The open triangles show the 24 individual fitted values. The solid triangle shows the log-average and the error bars are the standard deviation for the 24 individual fits, which are also written onto the figure. The average coefficient of variation over all data sets and the 24 replicate fits, <CV/dataset>, is also shown with its standard deviation. **Fig. 3B** shows the A∶B>A trajectories of 6 randomly chosen fits from the data for 30 mM digoxin transport, as an example. Four of the trajectories are on-target with the data, one is close and one is off-target. **Fig. 3C** shows the results for the 2^st^ round of 24 independent replicate fits, which was started as a fresh run with upper and lower bounds shown by the dashed box in Fig. 3A, together with appropriately reduced upper and lower bounds for the drug dependent kinetic parameters. The consensus average values, standard deviations and the ranges are given in Table 1. **Fig. 3D** shows the A∶B>A trajectories of 6 randomly chosen fits from the 2^nd^ round for 30 mM digoxin transport, like Fig. 3B. All six trajectories are on-target with the data and tighter than found in Fig. 3B for the 1^st^ round, hence the reduced range of fitted values.

This fitting run terminated when the maximum number of budgeted function evaluations was exceeded, which means that the global minimum had not been found, as defined by all particles converging to the same place. In order to visualize the end point of this fitting run, Fig. 3B shows 6 randomly chosen curves for the fits to the 30 mM digoxin data as an example. Four of the curves are clustered close to the data, while one diverges higher after about 12 hrs and one curve is significantly lower than the data. All 24 curves could be displayed, but the result is cluttered and yields the same basic conclusion, i.e. roughly a third of the fits were off target at this stage.

The CV for all 24 replicate fits was between 0.0259–0.0262, i.e. nearly identical. Fitting on a surface where the CV is nearly constant is inefficient. So rather than increasing the budget of the maximum number of function evaluations, we adjusted the upper and lower bounds to those shown by the dashed boxed area in Fig. 3A. In addition, we adjusted the upper and lower bounds for all the drug dependent kinetic parameters in the same way, i.e. including all fitted values and adding a small buffer zone, about 10–20%. This substantially reduced the volume of the parameter space to be explored in the 2^nd^ round, which started with a random dispersal of particles within the upper and lower bounds.

The 2^nd^ round of fits ended with the maximum number of function evaluation being exceeded, like the 1^st^ round. The result is shown in Fig. 3C. The average values of log_10_{*T(0)* (M)} = −3.0±0.1, log_10_{*k_1_* (M^−1^ s^−1^)} = 8.0±0.15 and the <CV/dataset> = 0.026, i.e. there was little change in the average values from the 1^st^ round. However, their standard deviations decreased about 3-fold in all measures from the 1^st^ round. In order to visualize this stage of the process, Fig. 3D shows 6 randomly chosen curves, which were not related to the 6 curves shown in Fig. 3B, since all fitting rounds were completely restarted. All 6 curves are clustered close to the data and show the convergence of the replicates to the same best-fit curve. None of the replicate fit values clustered near the new upper or lower bounds.

The upper and lower bounds were adjusted for a 3^rd^ fitting round, as was done for the 2^nd^ round. At the end of the 3^rd^ round, the average values of the fitted *T(0)* and *k_1_* did not change up to 3 significant digits. However, the average <CV/dataset> of the replicate fits increased slightly from the 2^nd^ round, suggesting that some of the upper and lower bounds for the other kinetic parameters were too restricted, despite being set outside the endpoints of the 2^nd^ round. Since the estimated values of *T(0)* and *k_1_* were essentially identical to those of the 2^nd^ round and well within experimental error of the individual experiments, we discarded the 3^rd^ round and continued the analysis of the fitted values from the 2^nd^ round. The primary function of the 2^nd^ round was to tighten the range for the drug dependent kinetic parameters to within around a factor of 3 or less, which allowed the simplest calculation of averages and standard deviations of the parameters themselves, not their log_10_ values.

### Consensus fits and ranges of the fits from the 2^nd^ Round

Table 1 contains the consensus fits for the kinetic parameters. The first column is the consensus for the association rate constant to P-gp from the membrane *k_1_* = (1±0.4)×10^8^ (M^−1^ s^−1^), to 1 significant digit. The whole range for *k_1_* in Fig. 3C for the 24 replicate fits was {0.6–2}×10^8^ (M^−1^ s^−1^) is shown underneath in curly brackets, {}, again to 1 significant digit. These numbers are drug independent.

The efflux active P-gp surface density is the next consensus fit shown in Table 1, in the units of P-gp/mm^2^. The average and standard deviation for the 24 replicate fits was 800±200 P-gp/mm^2^, while the range was {500–1300}, shown underneath in curly brackets.

### The fits for the drug dependent values for P-gp from the 2^nd^ round: *k_r_*, *k_2_* and *K_C_*

**Figure 4:**
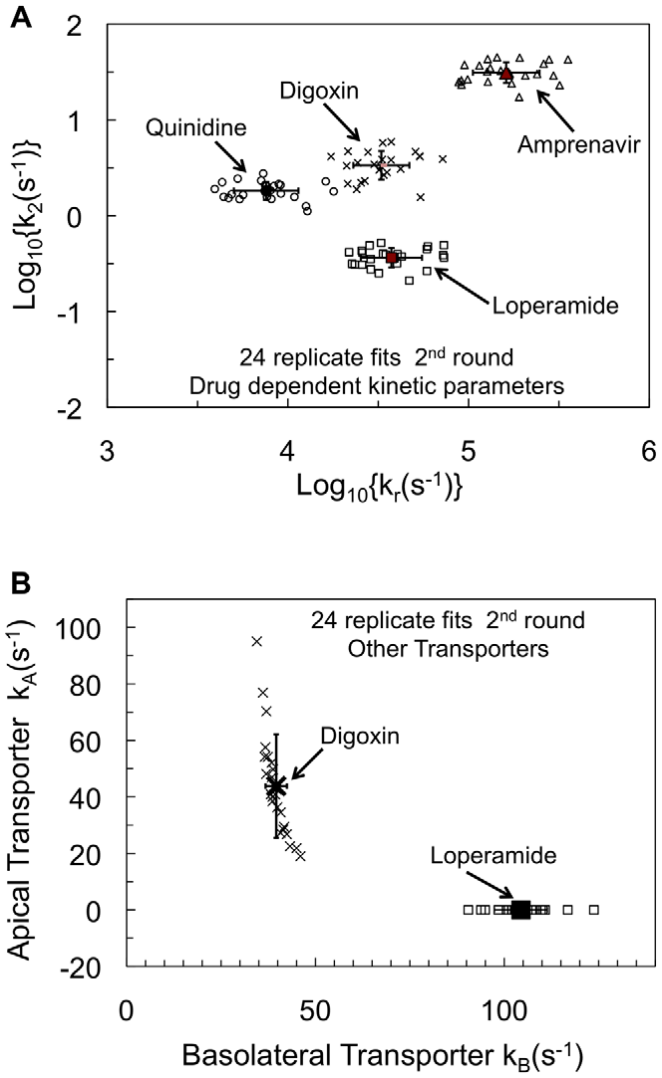
24 independent replicate fits from the 2^nd^ fitting round for drug dependent kinetic parameters. **Fig. 4A** shows the fitted values for *k_r_* and *k_2_* for each drug. The x- and y-axes show the upper and lower bounds for these fits. Like Fig. 3, the open symbols show the 24 individual fits for amprenavir (AMP, triangles), quinidine (QND, circles) and loperamide (LPM, squares) and digoxin (DGX, x). The closed symbols show the log-average with error bars showing standard deviations. **Fig. 4B** shows the fitted values for the loperamide basolateral transporter, *k_B_*, (LPM, squares) and for the digoxin basolateral and apical transporters, *k_B_* and *k_A_*, (DGX, x symbols). The closed symbols show the log-average with error bars showing standard deviations. The x- and y-axes show the upper and lower bounds for these parameters. The consensus average values, standard deviations and the ranges are given in Table 1.

We next looked at the fits for the drug dependent kinetic parameters. Fig. 4A shows the drug specific parameters {*k_r_*, *k_2_*} accompanying the 24 replicate values for {*T(0)*, *k_1_*} shown in Fig. 3C. The open symbols show the individual fitted values. The closed symbols show the consensus average values of *k_r_* and *k_2_* for each drug, error bars show the standard deviations. Table 1 shows the average and standard deviations calculation from the direct values, not their log_10_ values. The ranges from the 24 independent replicate fits are shown underneath the consensus average values in Table 1 in curly brackets.

Table 1 also shows the consensus binding constant, *K_C_* = *k_1_*/*k_r_*, for each drug to P-gp from the membrane, with standard deviation and the range obtained directly from the 24 independent replicate fits from Fig. 4A, i.e. not from the average *k_1_* divided by the average *k_r_*. The partition coefficients for the drugs was measured previously, using 0.1 mm diameter unilamellar liposomes whose compositions mimic, in a simple way, the lipids of the inner plasma membrane, *K_PC_*, the outer apical monolayer, *K_AO_*, and of the basolateral outer monolayer, *K_BO_* [23].

Another way we fit for the drug dependent kinetic parameters was to fix the values of *T(0)* and *k_1_* at their consensus values from Table 1 and fit all the drug dependent kinetic parameters using 12 independent replicate fits. We obtained replicate fitted values for {*k_r_*, *k_2_*}, all of which were essentially identical to the average values shown in Table 1, not shown. This showed that the average {*T(0)*, *k_1_*} value in Fig. 4A generated the average vector of drug dependent kinetic parameters.

### The fits for the other transporters

The final consensus values we need are those for the other basolateral and apical transporters. Fig. 4B shows the 24 independent replicate fits for the basolateral transporter required by loperamide transport kinetics (symbol squares) shown in the units of s^−1^ for a first order transporter [28], [29], [30]. These values are plotted on the *k_A_* = 0 line, since loperamide did not require an apical transporter, either here or previously [29]. The consensus average is <*k_B_*>≈100±7 s^−1^, to 1 significant digit, shown by the solid square with standard deviation, while the range was (90–125 s^−1^). This is shown in Table 1.

The steady-state values for the +GF120918 passive permeability coefficients of the other drugs are shown in the same column of Table 1. GF120918 completely inhibits both P-gp and the other transporters for loperamide and digoxin [23], [28], [29], [30]. If there are still other transporters in these cells which are not inhibited by GF120918, then the calculated +GF120918 passive permeability would include their contribution, in addition to the lipid bilayer permeability coefficient.

Fig. 4B also shows the 24 independent replicate fits for digoxin's basolateral and apical transporters, shown in the units of s^−1^ for the first order transporter (symbol x). The fits for the basolateral transporter are fairly tight. The consensus average is about <*k_B_*> = 40±3 s^−1^, with a range of {35–45 s^−1^}. This tightness of this fit was anticipated by the fit shown in Figure S4, where the basolateral transporter was essential to get a very good fit for the first 10 hrs of digoxin transport. The drift after 10 hrs that led to the addition of the apical transporter was not large and the wide range of 24 replicate fits for the apical transporter reflects this. The consensus average is about <*k_A_*> = 40±20 s^−1^, with a range of {20–95 s^−1^}. These values have been shown in Table 1, together with the relatively small +GF120918 steady-state passive permeability of digoxin.

### The other transporters for loperamide and digoxin are better fitted by a bidirectional mechanism compared with an active importer mechanism

We now address the question of whether the other transporters are more likely to be bidirectional or active transporters based upon best fitting of the data, since their identity is as yet unknown [29]. If these transporters are active, then they must be importers, since the problem shown in Fig. S4 is that without the basolateral transporter, not enough digoxin is getting into the cells from the basolateral chamber for P-gp to efflux into the apical chamber. Then, after 10 hrs, without the apical transporter, not enough of the digoxin effluxed by P-gp into the apical chamber was allowed to return into the cells. Basolateral or apical exporters cannot fix either of these problems.

**Figure 5:**
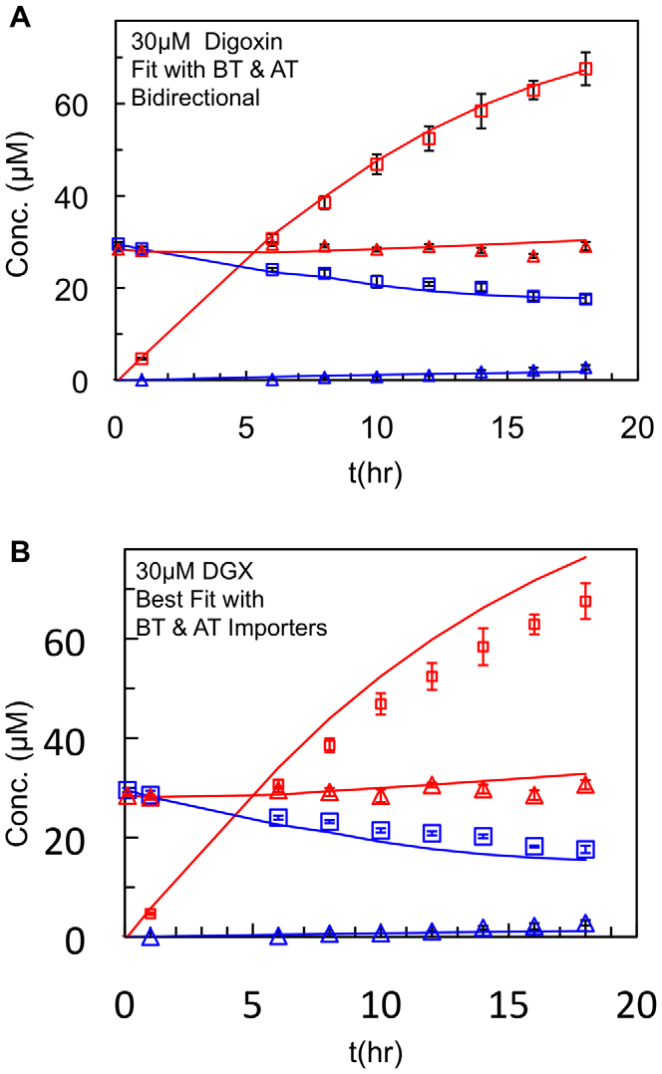
Fits of digoxin data with bidirectional or active importers. Fits of all the digoxin data with the assumption that the basolateral and apical transporters are bidirectional, **Fig. 5A**, or are active importers, **Fig. 5B**. The best fits for each mechanism are shown just for the 30 mM digoxin example, which is representative.

Fig. 5A shows the fit for 30 mM digoxin assuming that the basolateral and apical transporters are bidirectional, i.e. facilitate transporter. Using the previous algorithm [30], we could not obtain a fit for this particular data set, which is obviously fit well by the new Particle Swarm based algorithm. Next, we changed the basolateral and apical transporters to be importers only by setting the rate constants for transport out of the cells to zero. This automatically made the transporters active importers, without complicating the kinetic model unnecessarily with ATP hydrolysis kinetics. Of course, this did not affect P-gp. With {*T(0)*, *k_1_*} fixed at their consensus values in Table 1, the digoxin data was refit, including the *k_r_* and *k_2_* for P-gp. Fig. 5B shows the best fit for the importers with 30 mM digoxin. For all the digoxin data, the fits with importers are not as good as the fits with bidirectional transporters. The difference is not huge, so neither possibility can be completely rejected.

We refitted the loperamide data with an irreversible basolateral importer and found that the best fit <CV/dataset> = 0.023 over 25 datasets, as compared with <CV/dataset> = 0.020 for the bidirectional transporters. So, the bidirectional transporters yielded a better fit, as was the case for digoxin. However, if we refitted the loperamide allowing both basolateral and apical importers, then the fits were about the same as with just the basolateral bidirectional transporter. So, the loperamide transporters could be importers, but then but we would also require an apical importer for loperamide. We did not try other permutations.

### Fitting model data without error

We wanted to know whether the ranges of the {*T(0)*, *k_1_*} fits in Fig. 3 were due to experimental error. Using the consensus values obtained from the fits of experimental data, Table 1, we simulated model data without error for all the four drugs with concentrations: 0.01, 0.03, 0.1, 0.3, 1, 3, 10, 30 µM, while including 100 and 200 mM for amprenavir to reach P-gp saturation. If experimental error were the cause for the range in *T(0)* and *k_1_* fits, then the simulated data without error should yield a smooth funnel-like fitting surface with a common minimum at the parameter values used to simulate the data in the first place. The same fitting protocol used with the experimental data was followed.

For the model data without error, the 1^st^ and 2^nd^ rounds of fits for {*T(0)*, *k_1_*} with 24 independent replicate fittings, showed a similar broad range of fits just like the experimental data fittings in Figs. 3A and 3C, data not shown. The averages of the 24 replicate fits for the model data were essentially identical to the values used to simulate the data in the first place, data not shown. The <CV/dataset> of the model data fits were smaller by a factor of over 100-fold, as expected. So the range in the fits of the experimental data is not due to experimental error. The reason will be discussed below.

## Discussion

Using the transport data across a confluent monolayer of MDCKII-hMDR1 cells, we have constructed a molecular model of the P-gp membrane transport network based upon fitting the elementary rate constants of P-gp, the rate constants of other required transporters, as well as the passive permeability coefficients and partition coefficients [23], [29], [30]. All these components will be required to simulate the P-gp transport network for these and other drugs. Other cell lines or *in vivo* systems may well have other transporters, but our new kinetic analysis can identify their functional requirements for the observed substrate transport.

The ability to have simultaneous and relatively rapid fits over all relevant data sets overcomes the limitations of our previous method, while simplifying and clarifying the fitting process. For example, we can now survey data to find which drugs require other transporters and in which membrane, Table S1. If another transporter is required, we can survey the fitness of the potential mechanisms of that transporter, e.g. bidirectional/facilitated or active/importer or exporter, by determining which mechanism yields the best fit.

We will first discuss the sensitivity of the kinetic parameter fits, then we will discuss how the range of the fitted parameters is due primarily to compensation between kinetic parameters within this P-gp transporter network rather than experimental error and finally we will discuss how the values of the kinetic parameters explicate the biological function of P-gp and the other transporters.

### Sensitivity of the fits for the kinetic parameters

The fitted parameters from any multivariate nonlinear model are always subject to the question of parameter sensitivity, i.e. how much difference in the fit occurs with deviations from the “best” fit kinetic parameters. To answer this question we used independent replicate fits, i.e. a Monte Carlo approach. The maximum number of function evaluations was set at 12,000, which yielded a reasonable compromise between duration of a serial run, roughly a month, and compactness of endpoint parameter ranges.

At the end of the 1^st^ fitting round, Fig. 3A, which exhausted our budget for function evaluations, we determined new upper and lower bounds for all the parameters that encompassed their 24 endpoint values, with about a 10% buffer zone above and below for each fitted parameter. We could have restarted the fits from the endpoints of the 1^st^ round or simply started a new run from within the new boundaries. It was both simpler and more in keeping with the Particle Swarm philosophy to start fresh. This resulted in the endpoint of the 2^nd^ fitting round in about 2–3 weeks, Fig. 3C for *T(0)* and *k_1_*.

The average values of the efflux average surface density of P-gp, *T(0)*, and the association rate constant, *k_1_* did not change significantly between the 1^st^ to 2^nd^ rounds, Figs. 3A and 3C. The average fit got slightly better, <CV/dataset>. What really changed was the standard deviation of the average parameters, which is visualized by the difference between the six randomly chosen fits for the 30 mM digoxin A∶B>A data after each of the two rounds, Figs. 3B and 3D. At the end of the 1^st^ round, 4 of the 6 fits are close to target, 1 is ok and 1 is off target. At the end of the 2^nd^ round all 6 of the fits are on target and nearly identical. Thus the 2^nd^ round allowed the “laggard” fits to hit the data and tighten up a little, which accounts for a 3-fold decrease in the standard deviations for the fitted kinetic parameters.

Each of the final fits for {*T(0)*, *k_1_*} fits, Fig. 3C, had an associated vector of the drug dependent kinetic parameters, fitted simultaneously using just the drug specific datasets, including the other transporters for loperamide and digoxin. With the 2^nd^ round of fitting, all the kinetic parameters were within a 3-fold range, allowing us to simply average all these independent replicate fits, using their Cartesian (not logarithmic) values, and take their standard deviations, as shown in Table 1. The sensitivity of all these fitted parameters is very good, e.g. the standard deviations are <50% of the averages, Table 1. Given the complexity of the confluent cell monolayer and the rigor of our fitting, the error bars on the fitted parameters shows that the data is very tight.

Our next question about the fitting process was whether the {*T(0)*, *k_1_*} range was due to the experimental error in the data. To answer this question, model data without error was simulated using the consensus values of the kinetic parameters shown in Table 1. We fitted this simulated data following the same protocol as was used with the experimental data, including using 24 independent replicate fits. We found that the range of fitted values from these model data at the 1^st^ and 2^nd^ rounds was essentially the same as those for the experimental data shown in Fig. 3, i.e. no more compact. The average fitted values were essentially identical to the consensus values used to simulate the model data in the first place. So, the range of the fitted parameters is not due to experimental error.

### Fitted Parameter Compensation

The range of kinetic parameter fitted values is due primarily to compensation between kinetic parameters, yielding essentially the same transport trajectory, e.g. Fig. 3D. This compensation can occur only over a limited range of parameter values, since otherwise one of the parameters would be redundant. This means that there is no single rate-limiting step for transport at all substrate concentrations for any drug. One rate constant will dominate at low substrate concentrations, while other rate constants will dominate, in turn, at higher substrate concentrations, until transporter saturation was achieved. This is why the steady-state Michaelis-Menten equations, which assume a single rate-limiting step over the substrate concentration range, cannot give reliable estimates of *V_max_* and *K_M_* in terms of the elementary rate constants [27].

As rate determining dominance passes from one step in the transport process to another, there is a small zone of compensation. We illustrate this with a few examples. Table 1 shows that the value of *T(0)* ranges from 500–1300 P-gp per mm^2^, while the CV for all runs was essentially the same. So which kinetic parameters compensate to achieve essentially the same amount of transport within this zone?

This is shown using amprenavir, whose transport depends only on P-gp. The two fits showing the extreme values of *T(0)* out of the 24 runs, i.e. 500 and 1300 P-gp/mm^2^, have *k_r_* values of (1.4 and 1.9)×10^5^ s^−1^ and *k_2_* values of (45 and 17) s^−1^, respectively. *k_r_* is not much different, but the value of *k_2_* changes substantially. In fact, at the *T(0)* extremes, the product of *k_2_T(0)* has the values of {(2.25 and 2.21)×10^4^ P-gp/(mm^2^ s)}, i.e. they are essentially identical.

Previously, we found that that the product of *k_2_T(0)* was an important determinant for the fits [23]. Small variations in the fitted P-gp surface density can be compensated by inverse variations in the fitted efflux rate constant *k_2_*, such that their product remains essentially constant for a particular drug. Likewise, small variations in *k_1_* could be compensated by direct variations in *k_r_*, so that their ratio, *K_C_* = *k_1_*/*k_r_*, would remain essentially constant [23]. These two types of compensation is strong for drugs like amprenavir and quinidine which have only *k_r_* and *k_2_* to provide compensation for variations in *T(0)* and *k_1_*.

Loperamide and digoxin have the other transporters which can be involved in kinetic compensation. The range for loperamide's *k_B_* for its basolateral transporter was 90–124 s^−1^. For the fits showing the two extreme values, the values for *k_r_* are (2.3 and 7.2)×10^4^ s^−1^ and for *k_2_* are (0.31 and 0.39) s^−1^, respectively. Clearly, an increase in the value of *k_B_*, which would allow more drug into the cell to bind to P-gp, was compensated mostly by the increase in *k_r_*, the drug dissociation rate from P-gp back into the membrane, which would decrease the drug binding constant to P-gp. This yields about the same amount of drug bound P-gp and efflux of drug into the apical chamber. The same compensation pair is found with digoxin.

Compensation can involve more than just pairs of kinetic parameters. Fig. 4B shows that the apical digoxin transporter shows a large range in the fitted *k_A_* rate constant, from 95 to 22 s^−1^. At these two extremes, the *k_r_* values are (5.35 and 5.42)×10^4^ s^−1^, while *k_2_* values are (4.2 and 1.6) s^−1^. Before simply assigning the compensation to *k_2_*, since *k_r_* does not change much, we must first check whether *T(0)*, another compensatory partner of *k_2_*, is different For the extreme values of *k_A_*, *T(0)* has values of (1300 and 1100) Pgp/mm^2^, so the product of *k_2_T(0)* has the values of (5.5 and 1.8)×10^3^ P-gp/(s-mm^2^). The compensation is due mostly to these three kinetic parameters: *k_A_* versus the product of *k_2_T(0)*.

Compensation explains why model data without error does not have a funnel-like fitting surface. This points out why we cannot expect funnel-like fitting surfaces for multivariable transport networks, or probably any complex biochemical network.

### The values of the kinetic parameters

We have shown that fitted parameters are valid and their ranges make sense, so we can turn to what their numerical values imply. The values in Table 1 are different than those published previously using to the older algorithm. The difference is due to the fact that the Particle Swarm algorithm could fit all of our data simultaneously.

We start with the association rate constant *k_1_*. Since P-gp has its binding site in the inner apical monolayer [3], [5], [8] and all known P-gp substrates are amphipathic, it makes sense that the kinetically favored pathway to P-gp would follow the inner plasma membrane at the lipid lateral diffusion rate until it binds to P-gp. Our fitted *k_1_* is at the lipid lateral diffusion control range [23]. This would be a very rapid pathway, with the drug being able to diffuse through about half of inner monolayer of the plasma membrane, ∼20 mm in diameter, in 1–2 minutes. While P-gp probably evolved from a transporter of endogenous substrates, its current job in humans and other species appears to be the efflux of xenobiotic molecules, which come in all sizes and polar/nonpolar shapes [20], [33]. Thus, it also makes sense that P-gp's portal of entry to its binding site is large [8], which is also required for a large *k_1_*.

What about the efflux active P-gp surface density, *T(0)*? The term efflux active simply acknowledges our finding that the height and separation of the microvilli will determine which P-gp's can efflux drugs that can reach the apical chamber, where they are collected and assayed [23], [29]. Basically, in a random walk after efflux, only drug effluxed near the tips of the microvilli can be expected to reach the apical chamber in a timely fashion. The rest are adsorbed back into the microvilli membrane and recycled.

The efflux active P-gp surface density was fitted as 800±200 per mm^2^ or about 2×10^−2^ mg P-gp/cm^2^, assuming a molecular weight of 170 kD [34]. Rosenberg et al. [34] reported electron microscopy of P-gp proteosomes showing an average 10 nm diameter, including lipids. This means that P-gp will occupy roughly 100 nm^2^ of the apical membrane surface, i.e. close packing would yield roughly 10^4^ P-gp/mm^2^ or about 0.3 mg P-gp/cm^2^. Thus, our fitted value for the P-gp surface density occupies only about 8% of the available apical membrane surface area, which seems reasonable for an overexpressed membrane protein. None of the fitted values in the 2^nd^ round came close to the upper bounds for P-gp, Fig. 3C. A recently reported value of over 300 mg P-gp/cm^2^ for the hMDR1-MDCKII cells [35] cannot be correct, as it is about 1000-fold higher than close packing.

The selective pressure on P-gp appears to be maintenance of very broad substrate specificity, thus its binding constant to all xenobiotics should be relatively weak. Table 1 shows that 1/*K_C_* is in the mM range, with respect to dissociation back into the apical membrane. Thus, the binding to P-gp from the membrane is weak. However, the binding constant to P-gp relative to the cytosol is the inverse of the product of the substrate's partition coefficient and its binding constant, i.e. *K_D_* = 1/(*K_PC_.K_C_*). Our measured partition coefficients to liposome mimetics of the cell membrane monolayers (23) are greater than 100. These two parameters allow P-gp to bind and efflux substrates with micromolar cytosol concentrations.

Since the association rate, *k_1_*, is large and the binding constant *K_C_* is small, the dissociation rate constant back into the apical membrane, *k_r_* = *k_1_*/*K_C_*, must be large, as the fit shows in Table 1. The ratio of *k_r_*/*k_2_* estimates the number of bound substrate molecules that return to the apical membrane for each one effluxed into the apical compartment. From Table 1, this number ranges from about 3,000 for quinidine to 100,000 for loperamide. Thus, only the rare substrate occupying the P-pg binding site is actually effluxed, compared with the number dissociating back into the lipid bilayer.

This might appear inefficient usage of ATP, but P-gp's ATPase activity has not been measured to be higher than the maximal rate of FoF1, ∼100 s^−1^ [36], which we took for the upper bound for *k_2_* in our fitting. Protein ATPase activity may not be able to get much larger. However, this “inefficiency” vanishes when we consider that P-gp's job is to keep xenobiotics out of the cytosol, not out of the plasma membrane of the cell. This means that the efflux rate constant of P-gp is not competing against the return of drug to the membrane, but rather against permeation of the drug into the cytosol from the inner monolayer of the plasma membrane. Previously, we estimated that the rate of passive permeation of these substrates from the membrane into the cell cytosol were roughly 10 times slower than the smallest efflux rate constant [30], [37]. We can make more accurate estimates with our new values for the kinetic parameters. The equation needed is,
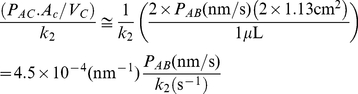
(2)where *P_AC_*.*A_C_*/*V_C_* is the passive permeability coefficient from inside the apical membrane to the cytosol times the area of the apical membrane divided by the volume of the cytosol. This is the first order rate constant, units of s^−1^, for the permeation from apical membrane interior to cytosol.

For simplicity, we use the entire apical membrane and cytosol of the confluent cell monolayer, rather than each individual cell. *P_AC_* is roughly equal to twice the A>B passive permeability, since passive permeability across membranes in series is like resistance, i.e. *P_AB_*≈1/(1*/P_AC_*+1/*P_BC_*) [12], [23], which accounts for the two barriers. Assuming they are equal gives *P_AC_* = 2×*P_AB_*. For the area of the apical membrane, we have used twice the plastic insert area, 2×1.13 cm^2^, simply to get the same passive permeability coefficient for the cell measurements, with two membranes, and the standard measurement using the 1-barrier equation [23]. Since the fit for *k_2_* also assumes efflux active P-gp surface area, the effect of the microvilli size and shape are roughly normalized out of this ratio.

For amprenavir, digoxin and quinidine, the Eq. (2) ratio is roughly 0.006, 0.004 and 0.08, respectively. Thus, P-gp keeps these drugs out of the cytosol with well over a 10-fold difference. For loperamide, the ratio is about 0.4–0.5, due to its much smaller fitted efflux rate constant. This suggests that loperamide is not as well cleared by P-gp from the cytosol as the other drugs. Further study with loperamide is warranted to understand why this is adequate for cell viability in different cell lines and tissues.

The range of values of the efflux rate constants, *k_2_*, covers nearly 2 orders of magnitude, i.e. from 0.4 s^−1^ for loperamide to 30 s^−1^ for amprenavir. Rank ordering of P-gp binding constants, *K_C_*, or equivalently the dissociation rate constants, *k_r_*, for the substrates is not monotonic with the rank ordering of efflux rate constants, showing that the molecular properties dominating these two reaction steps are not identical.

It is known that ATP hydrolysis by P-gp is required to efflux drugs [5], [9], [13], [20], [34]. The stoichiometry of ATPase activity to transport has been studied [5], but there is no definitive answer. The efflux rate constants we measure here will provide the best correlation between ATPase activity and efflux stoichiometry. The ATPase activity of P-gp depends on many factors and adding lipid to the purified protein increases the activity [37], [38], [39], [40], [41]. Many different values are reported, even for the same drugs.

It has been reported that the ATPase activity of purified P-gp reconstituted into proteosomes in 10 mM quinidine was about 4–6 mmol ATP hydrolyzed/min/mg P-gp [40]. This protocol gives consistent measurements for ATPase rates for other drugs [8], [22], [38]. This translates roughly to 10–15 s^−1^ ATPase activity compared with our fitted efflux rate constants of *k_2_* = 3 s^−1^, for both drugs. This suggests that the stoichiometry is about 3–5 ATPs hydrolyzed per quinidine molecule effluxed. This is significantly different from the commonly cited 1∶1 stoichiometry [5]. Obviously, many more cases will have to be examined before a conclusion can be reached. It may well be that the stoichiometry is not the same for all P-gp substrates.

The final kinetic parameters we need to discuss are the rate constants of the other transporters. For the loperamide basolateral transporter, the first order rate constant for the bidirectional mechanism was *k_B_* = 100 s^−1^ and no apical transporter was required. When the loperamide data was fitted using just the basolateral importer, the fit was worse. However, when we allowed both basolateral and apical importers for loperamide, the fits were basically the same as for just the bidirectional basolateral transporter. The fitted values for the importer mechanism were *k_A,IMP_* = 50 s^−1^ and *k_B,IMP_* = 100 s^−1^. Interestingly, the rate constant for the basolateral transporter did not depend on whether it was bidirectional or an active importer. This makes sense in that loperamide influx from the basolateral chamber was crucial to achieving good fits the data, which would be essentially the same whether the transporter was bidirectional or an active importer. The apical importer value of *k_A,IMP_* = 50 s^−1^ was compensated by a decrease in *k_r_*, to increase P-gp binding of loperamide and increase efflux to the apical chamber. *k_2_* did not change much. Thus, for loperamide, there are two reasonably clear alternatives for the other transporters.

When the other digoxin transporters were modeled as bidirectional/facilitated, there is the intriguing finding that both transporters having roughly the same rate constant, raising the possibility that it is the same transporter on both apical and basolateral plasma membranes. This may be unusual, but not impossible. When the digoxin transporters were modeled as importers only, the fit was not as good as for the bidirectional fits, Fig. 5. We do not yet have enough data for competition between digoxin and loperamide to deduce whether they compete for the same other transporters.

### Concluding Remarks

With this new fitting algorithm, we are now in a position to compare the kinetics of P-gp transport over a much wider range of substrates. This will include the kinetic identification of other transporters that affect the transport of any P-gp substrates, which will expand the P-gp transporter network. Our fitted rate constants make physical and evolutionary sense. The range of fitted values we show in Table 1 is due to the compensation partnerships between the kinetic parameters that define the P-gp transport network, rather than experimental error. This implies that transporter networks will not have a funnel-like fitting surface, but rather a relatively “flat” global minimum neighborhood, with respect to the coefficient of variation between the data and the best-fit curves. This rigorous analysis of P-gp function will enhance our understanding of how structure accomplishes this transport function.

Future work must address how the multiple substrate binding sites within P-gp [8] contribute to transport, whether these binding sites are competitive or uncompetitive, and/or cooperative, either positive or negative [11], [29], [42]. To fit cooperativity would double the number of elementary rate constants to be fitted. The new Particle Swarm based algorithm will facilitate rigorous surveys of all these mechanistic possibilities.

## Supporting Information

Figure S1 **Shows just the B>A transport, for clarity, during the first 6 hrs of transport.** The transport is much slower than that shown for amprenavir, due to digoxin's small +GF120918 passive permeability. The dashed lines are simply straight lines, not fits, showing that the transport data is linear. Fits for rate constants require curvature, such as seen with amprenavir after 2–3 hrs.
(TIF)

Figure S2 **Shows the transport over 18 hrs constructed from three separate experiments, wherein the concentration endpoints of Expt. 1, 0–6 hrs, were used for the initial concentrations for Expt. 2, 6–12 hrs.** Likewise, the concentration endpoints of Expt. 2, 6–12 hrs, were used for the initial concentrations for Expt. 3, 12–18 hrs. The three data sets were stitched together to create a continuous 18 hr transport curve which showed enough curvature to fit the kinetic parameters.
(TIF)

Figure S3 **Shows the culled dataset, reduced to 9 separate time points to accommodate the fitting program, wherein the initial time points with the straight data, Fig. S1, and then every other time point out to 18 hrs were omitted.**
(TIF)

Figure S4 **Shows the fitting for the other transporters.** While all datasets were fitted, only the fits for A∶B>A data are shown. The dotted black line shows the “best” fit using just P-gp. The fit requires maximal P-gp transport rate constants and is 50% too small. Adding a bidirectional apical transporter, AT shown by the dashed black line, makes no significant difference, since basolateral chamber is the donor here. Adding a bidirectional basolateral transporter, BT shown by the solid black line, allows a very good fit to the data up to about 8 hrs, after which time the fit overestimates the digoxin concentration in the receiver apical chamber. Adding bidirectional basolateral and apical transporters, BT & AT shown by the solid red line, allows a very good fit to the data over the entire time course, since the apical transporter allows digoxin to reenter the cytosol after P-gp efflux.
(TIF)

Table S1 **Effect of adding bidirectional transporters on fits.**
(DOC)

Text S1 (DOC)
