# Supplementary figures and images for: Fitting the Elementary Rate Constants of the P-gp Transporter Network in the hMDR1-MDCK Confluent Cell Monolayer Using a Particle Swarm Algorithm
Source: PLoS One. 2011 Oct 18;6(10):e25086. doi: 10.1371/journal.pone.0025086 (PMC3196501; doi:10.1371/journal.pone.0025086)

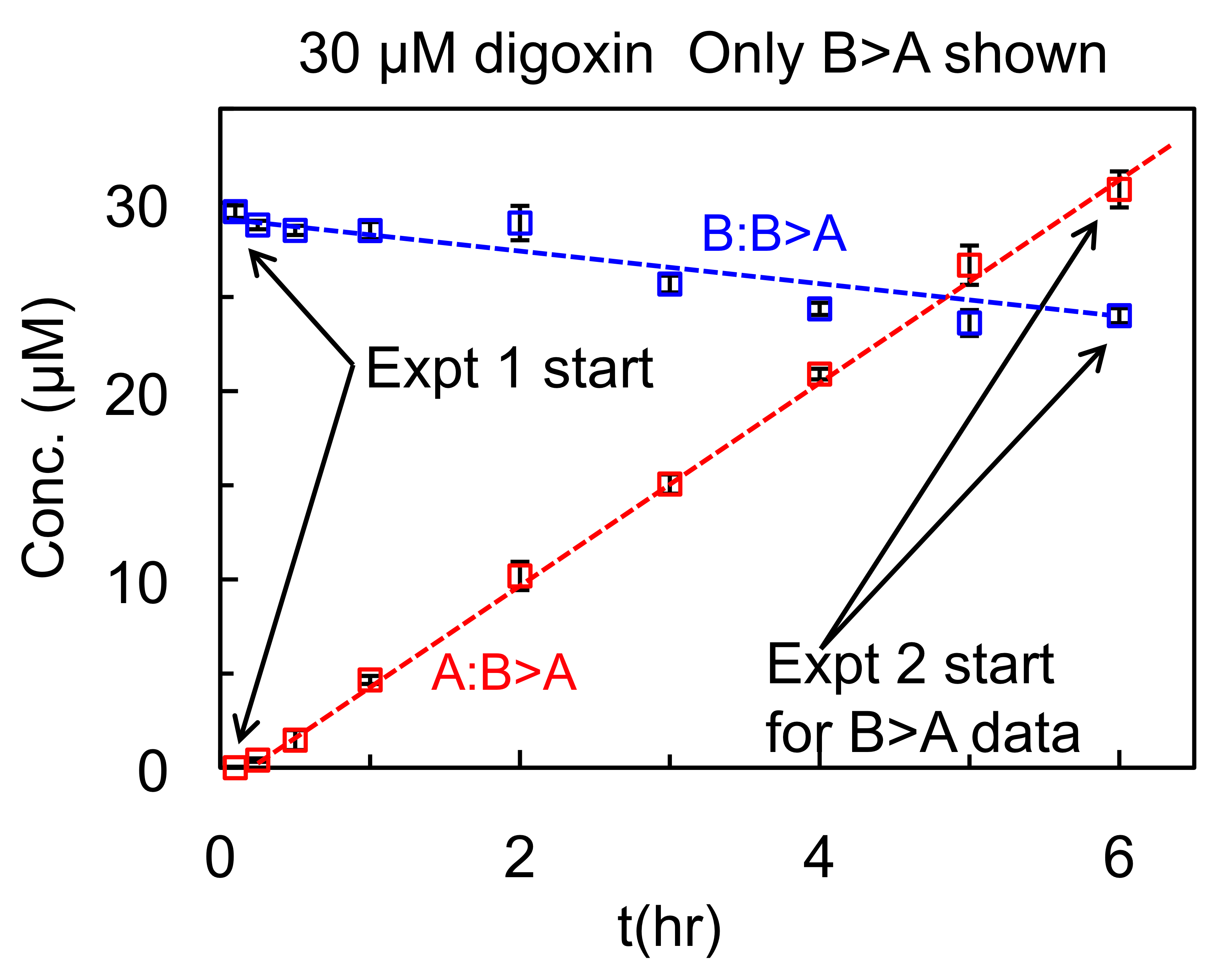

Supplement: Figure S1 — Shows just the B>A transport, for clarity, during the first 6 hrs of transport. The transport is much slower than that shown for amprenavir, due to digoxin's small +GF120918 passive permeability. The dashed lines are simply straight lines, not fits, showing that the transport data is linear. Fits for rate constants require curvature, such as seen with amprenavir after 2–3 hrs. (TIF) [file pone.0025086.s001.tif]

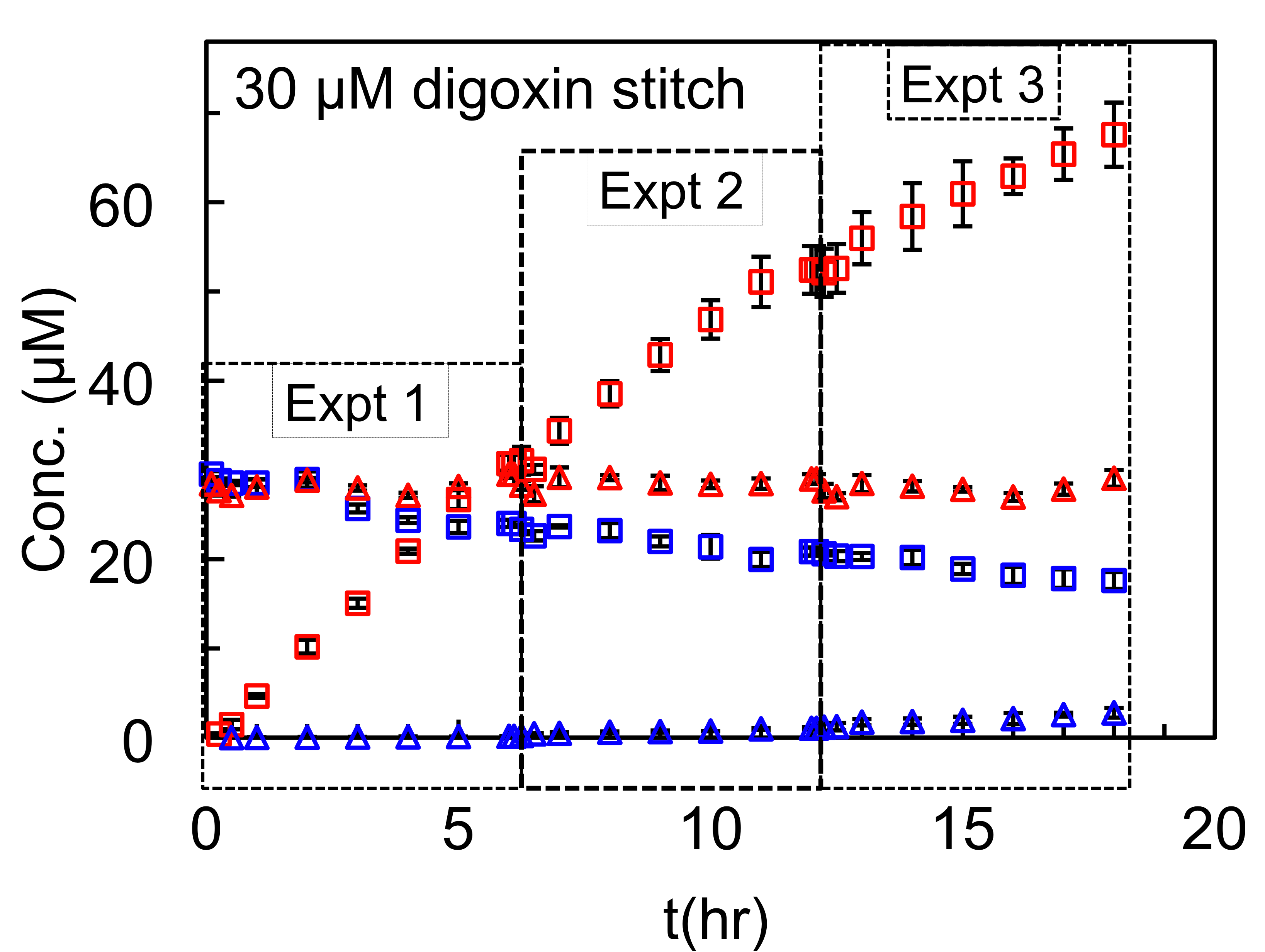

Supplement: Figure S2 — Shows the transport over 18 hrs constructed from three separate experiments, wherein the concentration endpoints of Expt. 1, 0–6 hrs, were used for the initial concentrations for Expt. 2, 6–12 hrs. Likewise, the concentration endpoints of Expt. 2, 6–12 hrs, were used for the initial concentrations for Expt. 3, 12–18 hrs. The three data sets were stitched together to create a continuous 18 hr transport curve which showed enough curvature to fit the kinetic parameters. (TIF) [file pone.0025086.s002.tif]

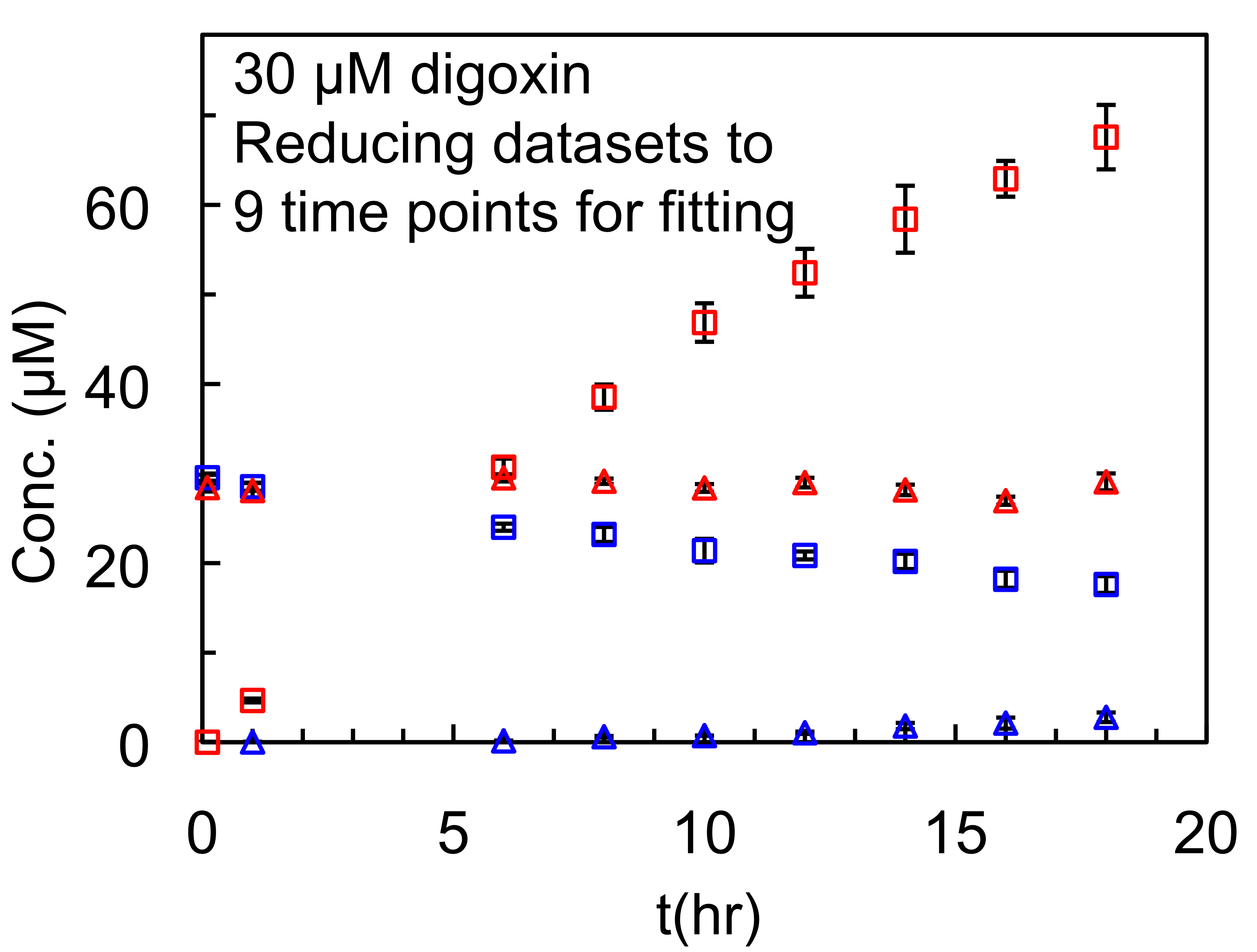

Supplement: Figure S3 — Shows the culled dataset, reduced to 9 separate time points to accommodate the fitting program, wherein the initial time points with the straight data, Fig. S1, and then every other time point out to 18 hrs were omitted. (TIF) [file pone.0025086.s003.tif]

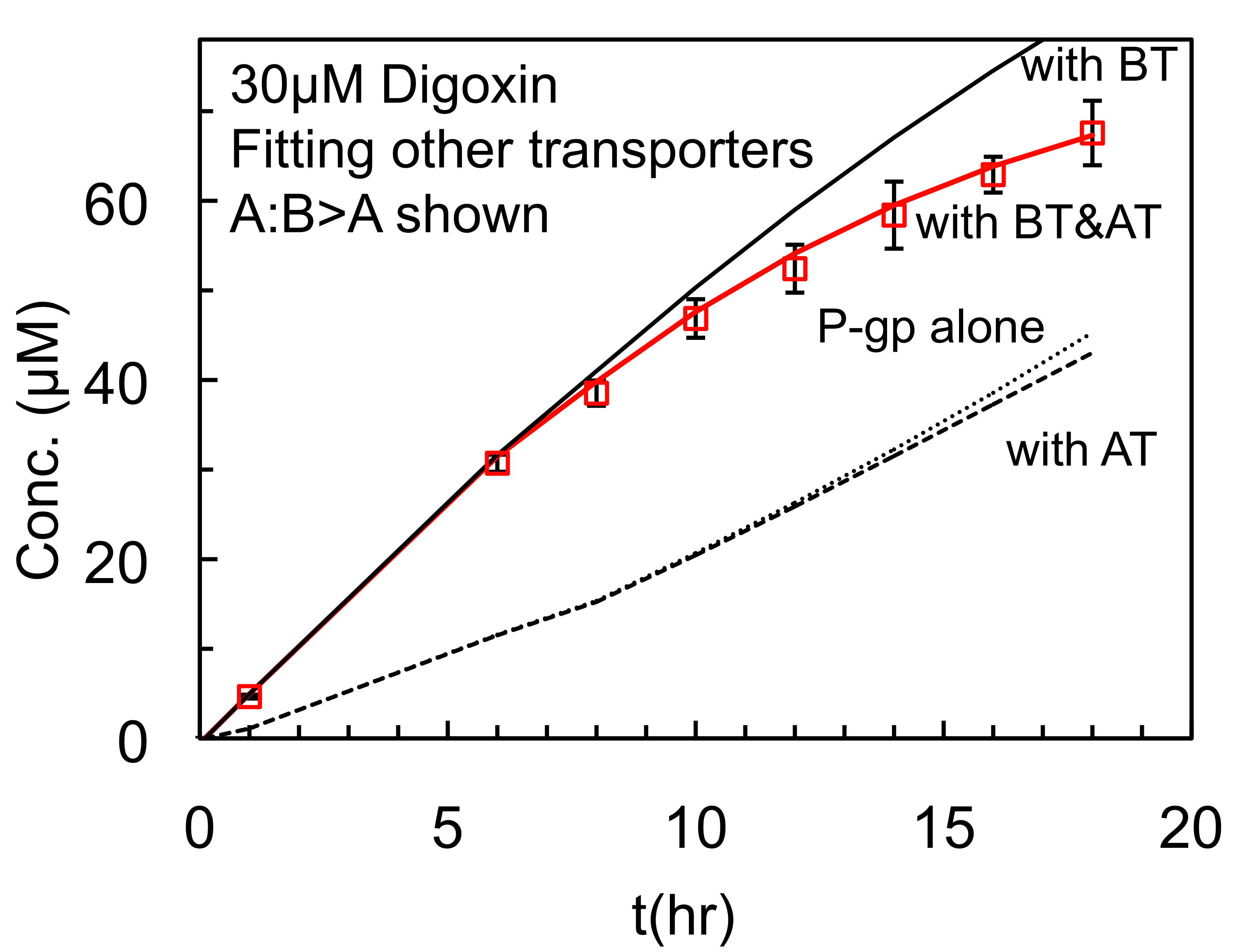

Supplement: Figure S4 — Shows the fitting for the other transporters. While all datasets were fitted, only the fits for A∶B>A data are shown. The dotted black line shows the “best” fit using just P-gp. The fit requires maximal P-gp transport rate constants and is 50% too small. Adding a bidirectional apical transporter, AT shown by the dashed black line, makes no significant difference, since basolateral chamber is the donor here. Adding a bidirectional basolateral transporter, BT shown by the solid black line, allows a very good fit to the data up to about 8 hrs, after which time the fit overestimates the digoxin concentration in the receiver apical chamber. Adding bidirectional basolateral and apical transporters, BT & AT shown by the solid red line, allows a very good fit to the data over the entire time course, since the apical transporter allows digoxin to reenter the cytosol after P-gp efflux. (TIF) [file pone.0025086.s004.tif]
