# Supplementary material for: Fitting the Elementary Rate Constants of the P-gp Transporter Network in the hMDR1-MDCK Confluent Cell Monolayer Using a Particle Swarm Algorithm
Source: PLoS One. 2011 Oct 18;6(10):e25086. doi: 10.1371/journal.pone.0025086 (PMC3196501; doi:10.1371/journal.pone.0025086)
Supplement: Table S1 — Effect of adding bidirectional transporters on fits. (DOC) [file pone.0025086.s005.doc]

Supporting Material: Tables

Table S1. Effect of adding bidirectional transporters on fits.

| Substrate | *k_r_* & *k_2_*^a^ | +AT ^b^ | +BT^c^ | +AT & BT ^d^ |
| --- | --- | --- | --- | --- |
| Amprenavir |  |  |  |  |
| <CV/dataset> | 0.029 | 0.026 | 0.025 | 0.025 |
| *k_r_* (s^-1^) | 2×10^5^ | 1×10^5^ | 2×10^5^ | 2×10^5^ |
| *k_2_* (s^-1^) | 30 | 100 | 30 | 30 |
| *k_A_* (s^-1^)^a^ |  | NU ^e^ |  | NU |
| *k_B_* (s^-1^) ^b^ |  |  | NU | NU |
| Quinidine |  |  |  |  |
| <CV/dataset> | 0.031 | 0.029 | 0.023 | 0.022 |
| *k_r_* (s^-1^) | 8×10^3^ | 1×10^4^ | 2×10^4^ | 2×10^4^ |
| *k_2_* (s^-1^) | 2 | 5 | 2 | 2 |
| *k_A_* (s^-1^) |  | NU |  | NU |
| *k_B_* (s^-1^) |  |  | NU | NU |
| Loperamide |  |  |  |  |
| <CV/dataset> | 0.044 | 0.042 | 0.020^f^ | 0.020 |
| *k_r_* (s^-1^) | 5×10^3^ | 6×10^3^ | 4×10^4^ | 4×10^4^ |
| *k_2_* (s^-1^) | 0.4 | 0.5 | 0.4 | 0.4 |
| *k_A_* (s^-1^) |  | NU |  | 2 |
| *k_B_* (s^-1^) |  |  | 100 | 100 |
| Digoxin |  |  |  |  |
| <CV/dataset> | 0.140 | 0.130 | 0.028 ^f^ | 0.021 ^f^ |
| *k_r_* (s^-1^) | "0" | 2×10^4^ | 8×10^4^ | 3×10^4^ |
| *k_2_* (s^-1^) | 90 | 30 | 2 | 3 |
| *k_A_* (s^-1^) |  | NU |  | 40 |
| *k_B_* (s^-1^) |  |  | 70 | 40 |

^a^ *T(0)* and *k_1_* are fixed at their consensus values, Table 1. Only *k_r_* and *k_2_* are fitted here.

^b^ An apical transporter, AT, is fitted along with *k_r_* and *k_2_*.

^c^ A basolateral transporter, BT, is fitted along with *k_r_* and *k_2_*.

^d^ Both an apical and a basolateral transporter, AT and BT, are fitted along with *k_r_* and *k_2_*.

^e^ NU: The transporter rate constants reported by the algorithm were not unique, since they had little impact on the fitted CV.

^f^ The shading indicates that these transporters are needed by these drugs to yield significantly better fits.
