# Supplementary material for: Fitting the Elementary Rate Constants of the P-gp Transporter Network in the hMDR1-MDCK Confluent Cell Monolayer Using a Particle Swarm Algorithm
Source: PLoS One. 2011 Oct 18;6(10):e25086. doi: 10.1371/journal.pone.0025086 (PMC3196501; doi:10.1371/journal.pone.0025086)
Supplement: Text S1 — (DOC) [file pone.0025086.s006.doc]

Supporting Material: Text

**Obtaining the data for fitting of kinetic parameters for digoxin.** Figures S1 – S4 show digoxin transport A>B and B>A over time across the MDCKII-hMDR1 confluent cell monolayer with 30 µM on the donor side initially. The experiments were conducted as three concurrent experiments, each 6 hrs long, for a total period of 18 hrs. The first experiment is shown in Fig. S1. The “initial concentration” for the 2^nd^ experiment was the “final concentration” of the 1^st^ experiment and so on and so forth, Fig. S2. The concentrations mentioned are the starting concentrations of digoxin in the donor chamber at the 1^st^ of the three experiments. The figures show the step-wise development of the digoxin data that was used for the Particle Swarm fitting algorithm to determine the kinetic parameters. Fig. S3 shows the culled data set used for the fitting program, as it is limited to 9 time points. Other culled sets gave the same or worse fits. The symbols show the data points with error bars showing the standard deviation of triplicate measurements.

**The other transporters for loperamide and digoxin are required.** Fig. S4 shows that the digoxin data fits best with both basolateral and apical transporters. Now, we address the quantitative aspects of “necessity” here. Table S1 compiles this information for each drug. The values for the efflux active surface density of P-gp, *T(0)*, and for the association rate constant, *k_1_*, were fixed at the drug independent consensus values shown in Table 1 (see article). The drug’s datasets were fitted with just *k_r_* and *k_2_*, denoted *k_r_* & *k_2_*, or by *k_r_* and *k_2_* plus an apical transporter (denoted +AT), or by *k_r_* and *k_2_* plus a basolateral transporter (denoted +BT) or by *k_r_* and *k_2_* plus both (denoted +AT&BT). Adding transporters changes fitted values for *k_r_* and/or *k_2_*.

Our simplest measure of whether the fit is improved by the addition of a transporter is the CV of the fit. Our primary interest here is proving the existence of the transporters. The fitted values are appropriate for the simplistic model we are using here.

For amprenavir, Table S1 shows that the addition of other transporters slightly reduces the CV. The best fit with the transporter in the apical membrane, +AT, reduces the CV by about 10% relative to the value for *k_r_* & *k_2_* only. However, to get this fit the value of P-gp’s efflux rate constant, *k_2_*, had to reach the upper bound of 100 s^-1^, fixed by the maximum rate measured for the F1Fo synthase [34]. This is a good reason to reject the requirement for an apical transporter. We have used NU in the table to denote a final fitted transporter value that was not unique, i.e. many other values would give essentially the same CV. Adding a basolateral transporter, +BT, or both, +AT&BT, made no significant difference on the amprenavir CV. So for amprenavir, there is no compelling evidence for any other transporter in these MDCKII-hMDR1 cells.

For quinidine, the addition of an apical transporter, +AT, yields a 7% reduction in the CV. However, the addition of a basolateral transporter, +BT, yields a 26% reduction in the CV, relative to *k_r_* & *k_2_* alone. At present, we believe this is inadequate to propose the need for another transporter, but more data and more rigorous statistics are needed.

For loperamide, the addition of an apical transporter to *k_r_* and *k_2_*, +AT, yields a 5% reduction in the CV. However, the addition of a basolateral transporter, +BT, yields about a 55% reduction in the CV, relative to *k_r_* & *k_2_* alone. This confirms our previous finding that loperamide requires a basolateral transporter [30]. We also see that adding an apical transporter to the basolateral transporter, +AT & BT, does not significantly improve the fit. Thus there is no requirement for an apical transporter for loperamide. This is a good example of our general experience that another fitting parameter in the “wrong place” does not make much difference to the fit. We shade this box in Table S1 to show the need for the basolateral transporter for loperamide.

For digoxin, the fit with only *k_r_* and *k_2_* is very poor, with a CV=0.140, while the addition of an apical transporter yields only a small reduction in the CV. However, the addition of a basolateral transporter yields nearly an 80% reduction in the CV relative to *k_r_* and *k_2_* alone. This is clearly significant and confirms our earlier finding that digoxin requires a basolateral transporter [30]. Interestingly, addition of the apical transporter with the basolateral transporter, +AT&BT, further reduces the CV by 25% relative to the basolateral transporter alone. Fig. S4 showed that this change was significant, since the deviation without the apical transporter increases monotonically over time after 10 hrs. This is an even better example of adding a fitting parameter in the wrong place does not improve the fit. The apical transporter alone does not make a better fit, since little digoxin reaches the apical chamber without the basolateral transporter. However, with the basolateral transporter, the apical transporter significantly improves the fit. We shade both the +BT and the +BT & AT boxes to indicate these transporters are both required.

Legend for Supplementary Figures

**Fig. S1** shows just the B>A transport, for clarity, during the first 6 hrs of transport. The transport is much slower than that shown for amprenavir, due to digoxin’s small +GF120918 passive permeability. The dashed lines are simply straight lines, not fits, showing that the transport data is linear. Fits for rate constants require curvature, such as seen with amprenavir after 2-3 hrs.

**Fig. S2** shows the transport over 18 hrs constructed from three separate experiments, wherein the concentration endpoints of Expt. 1, 0-6 hrs, were used for the initial concentrations for Expt. 2, 6-12 hrs. Likewise, the concentration endpoints of Expt. 2, 6-12 hrs, were used for the initial concentrations for Expt. 3, 12-18 hrs. The three data sets were stitched together to create a continuous 18 hr transport curve which showed enough curvature to fit the kinetic parameters.

**Fig. S3** shows the culled dataset, reduced to 9 separate time points to accommodate the fitting program, wherein the initial time points with the straight data, Fig. S1, and then every other time point out to 18 hrs were omitted.

**Fig. S4** shows the fitting for the other transporters. While all datasets were fitted, only the fits for A:B>A data are shown. The dotted black line shows the “best” fit using just P-gp. The fit requires maximal P-gp transport rate constants and is 50% too small. Adding a bidirectional apical transporter, AT shown by the dashed black line, makes no significant difference, since basolateral chamber is the donor here. Adding a bidirectional basolateral transporter, BT shown by the solid black line, allows a very good fit to the data up to about 8 hrs, after which time the fit overestimates the digoxin concentration in the receiver apical chamber. Adding bidirectional basolateral and apical transporters, BT & AT shown by the solid red line, allows a very good fit to the data over the entire time course, since the apical transporter allows digoxin to reenter the cytosol after P-gp efflux.
